# Finite Element Analysis of Stress Distribution in Canine Lumbar Fractures with Different Pedicle Screw Insertion Angles

**DOI:** 10.3390/vetsci12070682

**Published:** 2025-07-19

**Authors:** Ziyao Zhou, Xiaogang Shi, Jiahui Peng, Xiaoxiao Zhou, Liuqing Yang, Zhijun Zhong, Haifeng Liu, Guangneng Peng, Chengli Zheng, Ming Zhang

**Affiliations:** 1 Teaching Veterinary Hospital, College of Veterinary Medicine, Sichuan Agricultural University, Chengdu 611130, China; 2 Sichuan Wolong National Natural Reserve Administration Bureau, Wenchuan 623006, China; 3 Chengdu Center for Animal Disease Prevention and Control, Chengdu 610041, China; 4 Sichuan Institute of Musk Deer Breeding, Sichuan Institute for Drug Control, Chengdu 611130, China; 5 College of Animal Science, Sichuan Agricultural University, Chengdu 611130, China

**Keywords:** canine lumbar spine, finite element analysis, pedicle screw fixation, insertion angle, biomechanical stability

## Abstract

Pedicle screw fixation is a critical technique for stabilizing lumbar fractures in canines, yet the biomechanical implications of insertion angles remain underexplored. This study aims to identify optimal screw trajectories by analyzing stress distribution and deformation patterns in beagle lumbar segments (L6-L7) using finite element analysis (FEA). A 3D finite element model was reconstructed from CT scans of a healthy beagle, incorporating cortical/cancellous bone, intervertebral disks, and cartilage. Pedicle screws (2.4 mm diameter, 22 mm length) were virtually implanted at angles ranging from 45° to 65°. A 10 N vertical load simulated standing conditions. Equivalent stress and total deformation were evaluated under static loading. The equivalent stress occurred at screw–rod junctions, with maxima at 50° (11.73 MPa) and minima at 58° (3.25 MPa). Total deformation ranged from 0.0033 to 0.0064 mm, with the highest at 55° and the lowest at 54°. The 58° insertion angle demonstrated optimal biomechanical stability with minimal stress concentration, with 56–60° as a biomechanically favorable range for pedicle screw fixation in canine lumbar fractures, balancing stress distribution and deformation control. Future studies should validate these findings in multi-level models and clinical settings.

## Simple Summary

Back injuries, especially fractures in the lower spine, are common in dogs. Surgeons often use special screws to stabilize these fractures, but the best angle to place these screws is not well understood. In this study, we used computer simulations to test how different screw angles affect stress and movement in a dog’s spine. We created a 3D model of a beagle’s lower back from CT scans and tested screws inserted at angles between 45° and 65°. Our results showed that screws placed at 58° caused the least stress on the spine, while angles between 56° and 60° provided the most stable fixation. This information can help veterinarians perform safer and more effective surgeries for dogs with spinal fractures. Future research should test these findings in real surgeries and different dog breeds.

## 1. Introduction

Common lumbar spinal disorders in canines encompass a variety of conditions including intervertebral disk herniation, vertebral fractures, neoplastic lesions affecting the spinal cord and nerve roots, and acute spinal cord injuries [1,2]. Among these pathologies, vertebral fractures or dislocations of traumatic etiology account for approximately 7% of cases, with lumbosacral region injuries being particularly prevalent [2]. In addition, when genetic or congenital ligament structural instability weakens the support function of the spine, potential pathological processes may lead to impaired bone integrity, which in turn can cause pathological fractures [3]. These lumbosacral fractures and dislocations constitute 39% of all spinal pathology cases documented in veterinary practice [4], which may lead to motor dysfunction, and urinary and fecal incontinence, seriously affecting quality of life in the animal.

Surgical intervention for lumbar fractures in canines is a method for achieving an anatomical reduction in the fractured vertebral segment, coupled with spinal cord decompression, contingent upon the neurological status and the extent of the traumatized region. Research has shown that there is a significant correlation between the treatment effect of canine spinal fractures and anatomical features [5]. In contrast to human spinal anatomy, canine vertebral dislocations and diverse fracture patterns typically present in recumbency, necessitating that pedicle screws and vertebral body implants be strategically positioned to traverse the maximal transverse diameter of the vertebral body [6]. This anatomical consideration enables secure fixation across adjacent vertebrae. The L6 segment is particularly susceptible to lumbar fractures in dogs, whereas the L7 segment has been identified as the most favorable site for pedicle screw fixation.

Finite element analysis (FEA) is a digital calculation method that uses computer-aided design to construct research models, simulate mechanical processes, and conduct mechanical analysis. The basic idea of FEA is to divide a certain structure into a number of small elements, assigning various mechanical properties (such as the elastic modulus and Poisson’s ratio), to establish a matrix equation under loaded conditions for calculating the stress–strain relationship influenced by external forces, pressure, and other factors [7]. In the field of biomechanics, FEA can effectively simulate the mechanical response under complex physiological conditions, being especially suitable for the mechanical performance evaluation of the skeletal system [8]. Moreover, computer simulation can effectively reduce the use of experimental animals, in line with the 3R principle [9]. In human medicine, three-dimensional finite element analysis has been employed to enhance the accuracy of surgical approach evaluation and intraoperative planning in spinal surgery. Within the neurosurgical field, drill guide template technology has been developed for precise guidance during spinal fixation screw placement [10]. Previous studies have proposed a pedicle screw insertion angle range of 45° to 65° for canine lumbar fixation [11]; however, the specific optimal angles within this range for canine clinical applications remain unexplored.

Therefore, this study, using beagle dogs as experimental subject, initiated a three-dimensional (3D) model of the L6 and L7 lumbar segments in beagle dogs, derived from CT scan data. Subsequently, a finite element fracture model was constructed using Mimics, Geomagic, and Solidworks software. This model was then imported into Ansys for FEA to ascertain the distribution of equivalent stress and total deformation when pedicle screws were inserted at angles ranging from 45° to 65°. This analysis aims to provide a theoretical foundation for the biomechanical investigation of lumbar fractures in canines.

## 2. Materials and Methods

### 2.1. Ethics Statement

This study was approval by the Sichuan Agricultural University Animal Ethical and Welfare Committee with the approval number 20240221. All the animal procedures were conducted in strict accordance with the National Standards for Laboratory Animal Welfare (GB/T 35892-2018, China) [12], designed to minimize discomfort, including standardized anesthesia monitoring and post-procedural care.

### 2.2. CT Imagine Collection of Beagle Dog

A 2-year-old male beagle dog (body weight: 14.2 kg) was selected for this study, provided by the Sichuan Science and Technology Resources Sharing Platform of Beagle Dog Breeding and Experimental Technology Service. Anesthesia was induced with an intravenous injection of Zoletil^®^ 50 (tiletamine-zolazepam, Virbac, France) at a dose of 10 mg/kg, and maintained with isoflurane (1–2% in oxygen) to ensure stable vital signs (heart rate: 80–100 bpm; respiratory rate: 15–20 breaths/min; rectal temperature: 38.0–39.0 °C) during scanning.

The dog was positioned in the supine position for CT scanning in Chengdu Ultrasound Imaging Center Co., Ltd., Chengdu, China (model uCT503e, featuring 40 rows and 40 layers). The CT scan settings were as follows: tube voltage, 120 kV; tube current, 250 mA; slice thickness, 3 mm; rotation time, 1.00 s; pitch, 0.6250; reconstruction matrix, 512 × 512; reconstruction slice thickness, 1 mm; bone window width, 2600 HU; window level, 800 HU; soft tissue window width, 300 HU; and window level, 40 HU. These settings were similar to those applied in our previous study [13].

The CT images were reviewed by a veterinary radiologist board-certified by China Veterinary Association to confirm the absence of spinal abnormalities (e.g., congenital deformities, fractures, or disk herniation) and ensure the clarity and completeness of L6-L7 segments.

### 2.3. Three-Dimensional Reconstruction of the Lumbar Spine

The 3D reconstruction tasks were performed on a personal computer equipped with an Intel(R) Core(TM) i5-8300H CPU operating at 2.30 GHz, with 16.0 GB of RAM, and running the Windows 10 Professional operating system, manufactured by ASUS Computer Inc. The 3D reconstruction and modeling were performed using Mimics 21.0, developed by Materialise in Vallda, Sweden. The DICOM format CT images were imported into Mimics 21.0 software for 3D reconstruction.

In Mimics 21.0, the threshold range for bone was set between 226 and 3071 HU, consistent with previous study [14]. After threshold selection, Mimics identified all regions within the lumbar spine. A base mask was created, and the “Measure” command was used to measure the length of the lumbar spine. Irrelevant regions (e.g., rib fragments, soft tissue) were manually erased using the “Edit masks” function, and voids within the vertebral body (due to partial volume effects) were filled with the “Close Holes” tool (hole diameter < 2 mm). To refine the segmentation of disconnected bony structures (e.g., articular processes), “Region growing” was applied with a seed point placed at the center of the L6 vertebral body, using a threshold tolerance of ±10 HU to ensure consistent inclusion of cancellous bone.

### 2.4. Smoothing and Repairing of the Bone Models

The 3D model was neatly computed using the “Compute Component” command. To ensure accuracy in finite element analysis, the model quality was set to “Optimal.” Surface smoothing was performed using the “Remesh” operation in the 3-matic module of Mimics. The “wrap” and “smooth” functions were applied, with the smooth coefficient set to 0.3 (balanced to preserve anatomical details while reducing noise). The model was then exported into STL format and imported into Geomagic Wrap 2017 (3D Systems, Rock Hill, SC, USA) for further smooth processing.

Cortical and cancellous bone were separated based on CT value thresholds (cortical bone: >800 HU; cancellous bone: 226–800 HU) and imported into SolidWorks 2021 (Dassault Systèmes, Vélizy-Villacoublay, France) for solidification. The L6 and L7 vertebrae were modeled as separate components and aligned at the global origin. An intervertebral disks (L6-L7) and articular cartilage were designed using anatomical measurements. The intervertebral disk and cartilage were created using sketching, copying, splitting, combining, and intersecting operations. The L6 segment was aligned vertically, and a fracture surface was cut perpendicular to the long axis of the vertebral body.

### 2.5. Assembly of the Lumbar Fracture Internal Fixation System

Pedicle screws were selected for internal fixation at different insertion angles. The screws were designed based on clinical standards and the size of the beagle. Four pedicle screws were modeled in Solidworks, each with a cap diameter of 4 mm, screw diameter of 2.4 mm, and total length of 22 mm [15]. The screws were inserted into the lumbar model at angles ranging from 45° to 47°, 50°–60° with 1° intervals, 63°, and 65°.

The assembled model was imported into Ansys, and each component was set as a separate part. Surface mesh inspection was performed to remove and manually repair deformed, overlapping, or disconnected meshes.

### 2.6. Finite Element Parameter Assignment and Loading

The cortical bone, cancellous bone, annulus fibrosus, nucleus pulposus, and ligaments were treated as linear elastic materials. The material properties were simplified to isotropic and linear elastic to improve computational efficiency. The pedicle screws and rods were made of titanium alloy (Ti-6Al-4V), with material parameters derived from previous studies. The material parameters are listed in Table 1.

### 2.7. Finite Element Analysis

For this study, a vertical load of 10 N was applied to simulate normal standing or walking conditions. The contact between the lumbar spine and implants was defined as fixed, with no sliding allowed. The intervertebral disk and vertebral body contacts were set as bonded, and the facet joints were defined as non-separable in the vertical direction but free to move horizontally. A 10 N force was applied to the spinous process, and the distal end of the lumbar spine was set as a fixed support.

The 3D finite element model was imported into Ansys. The equivalent stress and total deformation were calculated to compare the stress distribution and deformation in the lumbar spine with pedicle screws inserted at different angles under a 10 N vertical load.

## 3. Results

### 3.1. Model Establishment Results

**Figure 1:**
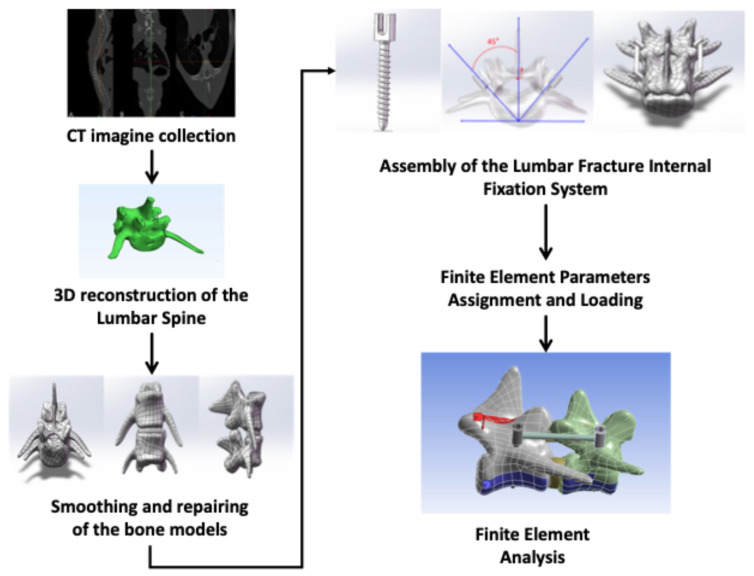
Scheme of 3D model and finite element analysis.

A high-fidelity 3D model of the beagle dog’s L6-L7 lumbar segments was successfully reconstructed from CT scan data, with clear visualization of anatomical details including the vertebral bodies, pedicles, transverse processes, spinous processes, intervertebral disk (L6-L7), and articular cartilage (Figure 1).

The pedicle screw model was designed with a cap diameter of 4 mm, a screw diameter of 2.4 mm, and a total length of 22 mm, featuring a threaded arc length of 106.56 mm. To systematically evaluate the influence of insertion angles on biomechanics, 15 finite element models were constructed, each with pedicle screws implanted at distinct angles relative to the vertebral sagittal plane: 45°, 47°, 50–60° (at 1° intervals), 63°, and 65°. Mesh quality assessment for each model (Table 2) showed node counts ranging from 161,757 to 168,027 and element counts ranging from 83,010 to 87,710, indicating high mesh quality suitable for subsequent FEA.

### 3.2. Equivalent Stress at Different Pedicle Screw Implant Angles

**Figure 2:**
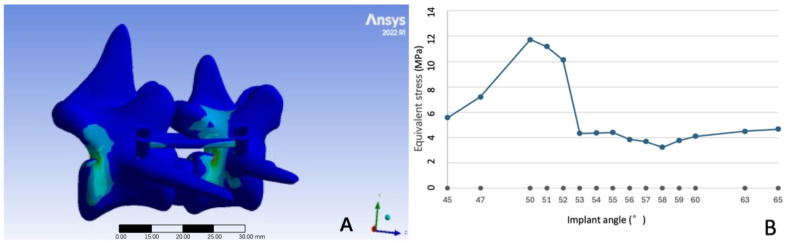
Equivalent stress at different pedicle screw insertion angles. (**A**) Distribution map of equivalent stress at 45°implant angle; (**B**) equivalent stress at different implant angles.

Under a 10 N vertical load, the equivalent stress (von Mises) distribution across the L6-L7 fixation system varied significantly with pedicle screw insertion angles (Figure 2A). Consistent with biomechanical principles, stress concentration was most prominent at two key locations, (1) the screw neck (the junction between the screw cap and shaft) and (2) the interface between the screw cap and the fixation rod, which are recognized as potential high-risk regions for implant fatigue failure in spinal fixation systems.

Quantitative analysis revealed that peak equivalent stress across all angles ranged from 3.2454 MPa to 11.7300 MPa (Figure 2B), with the highest value observed at 50° (11.7300 MPa) and the lowest at 58° (3.2454 MPa). Notably, stress values fluctuated substantially between 45° and 55° (range: 7.82–11.73 MPa), whereas angles between 56° and 60° showed relative stability (3.25–5.12 MPa).

### 3.3. Total Deformation at Different Pedicle Screw Insertion Angles

**Figure 3:**
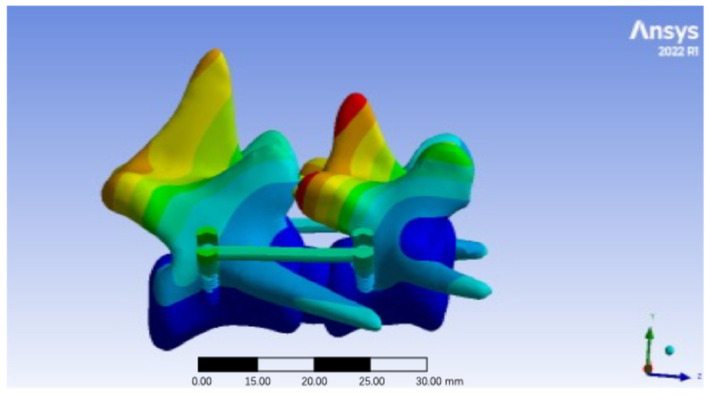
Total deformation distribution diagram of pedicle screw implantation in the lumbar spine (45°).

Total deformation (cumulative displacement) of the L6-L7 fixation system under 10 N loading was primarily localized to the intervertebral disk (L6-L7) and articular cartilage of the facet joints, consistent with their role as compliant structures in the spinal column (Figure 3).

Quantitative analysis of peak total deformation (Table 3) showed a range of 0.0033 mm to 0.0064 mm across all angles. The highest deformation was recorded at 55° (0.0064 mm), primarily concentrated in the posterior annulus fibrosus of the L6-L7 disk, suggesting increased tensile stress on the disk at this angle. Conversely, the lowest deformation was observed at 54° (0.0033 mm), with minimal displacement in both bony and soft tissue structures, indicating enhanced stability of the fixation system. Among them, angles between 51° and 54° consistently exhibited low deformation (<0.0040 mm), while angles at the extremes of the tested range (45°, 55°) showed higher displacement (>0.0055 mm), highlighting a potential relationship between insertion angle and system stability.

## 4. Discussion

Finite element analysis has unique advantages in orthopedic biomechanics, including the following: (1) non-invasive modeling, which allows simulation of complex biomechanical behaviors without the need for physical specimens or animal trials; (2) stress–strain analysis that can predict stress distribution in bones, implants, and soft tissues, helping identify potential failure points; and (3) pre-surgical planning and virtual testing that helps surgeons evaluate different fixation strategies as well as implant performance under extreme conditions before real-world application [16,17]. Some canine lumbar biomechanical studies have existed. Yuki Kikuchi et al. [18] developed L1-L2 finite element models from canine CT data, applying 0.2–2 Nm incremental loads to demonstrate functional spinal unit stabilization. Their parallel mechanical testing and FEA of L1-L2 and L5-L6 segments showed strong correlation between computational predictions and experimental results. Tailong Yu et al. [19] designed a biodegradable dynamic stabilization system for interfaced lumbar fusion, demonstrating comparable fusion outcomes and biomechanical performance to those of rigid fixation with safe in vivo degradation. However, our study is the first study using the FEA method for determining the best angle for pedicle screw insertion in L6-L7.

During normal standing or walking, dogs primarily experience vertical downward pressure on the lumbar spine due to their weight being transmitted through the limbs. TAE-HONG LIM et al. [20] conducted a comprehensive biomechanical evaluation of the intact L6-L7 functional spinal unit. Their model predicted key parameters including flexion angle, axial stiffness, and facet joint contact forces. The study established that applying a load equivalent to 7% of canine body weight represents a physiologically reasonable loading condition for lumbar spine analysis. Given that the present experiment primarily investigates stress distribution during normal walking and standing states rather than overload conditions, the vertical loading force was set at an average of 10 N for beagles (7% of 14 kg), with the direction oriented vertically downward. A frictional contact interaction was defined between the lumbar vertebrae and the implant, which was modeled as a fixed interface.

In FEA, equivalent stress characterizes the internal resistance of a material to deformation under external loading. The displacement reflects the relative motion between the implant and bone tissue, serving as a critical indicator for evaluating the stability of the internal fixation system. Total deformation value refers to the microscopic displacement of bone tissue under mechanical loading at fracture sites, which serves as an indicator for assessing bone stability [21]. A study [22] reported that titanium alloy implants undergo irreversible deformation at a stress of 450 MPa, with fracture risks exceeding 600 MPa. Our results demonstrated that the greatest implant stress was 11.73 MPa, with fracture risks being much lower. Comparative analysis of maximum equivalent stress nephograms across internal fixation models revealed consistent stress distribution patterns in pedicle screws, with maximum axial displacement occurring proximally at loading sites and gradually decreasing distally. The 51°–54° and 59°–65° insertion angles showed lower displacement magnitudes compared to overall values, with peak total deformation ranging between 0.0033 and 0.0064 mm. These findings illustrated that the degree of postoperative micro-motion of the pedicle screw fixation end is within an acceptable range, reducing the risk of surgical failure caused by displacement [23].

Although the FEA results are all within the acceptable range of lumbar spine biomechanics, notable abrupt deformations or stress fluctuations were observed in response to specific angular deviations. For example, the maximum intervertebral disk deformation (0.0064 mm) occurred at 55°, while the minimum deformation (0.0033 mm) was observed at 54°; the stress at the L6 fracture site reached 8.7 MPa at 50°, but decreased to 3.5 MPa at 58°. Two potential mechanistic explanations may underlie these abrupt mechanical changes: (1) Anatomical influence of the lumbar spine: lumbar vertebral bodies exhibit an irregular elliptical geometry. During screw implantation, as the trajectory approaches the elliptical apices of the vertebral body, a mechanical threshold is reached, triggering dramatic stress–deformation responses. (2) Bone tissue transition with angular variation: alterations in screw angulation led to a shift in contact from cancellous to cortical bone within the transverse process, directly modulating mechanical loading profiles and stress distribution patterns. Although vertebral displacement represents a primary determinant of crack initiation and propagation prior to fracture, minor deformations may be fixed by Wolff’s law [24]. During screw insertion, cancellous bone within pedicles becomes compacted around screws, creating cavities. Following fracture healing and implant removal, trabecular bone within these cavities undergoes thickening under mechanical stimulation, leading to displacement variations as trabeculae reorganize into denser, more regular patterns [25]. Additionally, there are varying ranges of macroscopic yield strain before bone failure, while bone strain also functions as a mechanical signal triggering bone remodeling during healing processes [26,27]. Therefore, postoperative rehabilitation should include appropriate activity selection, as controlled lumbar displacement may facilitate fracture healing [28].

Our results indicate that optimal screw trajectories in beagles target high-density regions at the ventrobasal pedicle–vertebral junction. For lumbar vertebral fractures, bilateral pedicle screw fixation systems can significantly suppress abnormal intervertebral motion, and this rigid fixation method helps maintain postoperative spinal mechanical stability. While L7 permits similar implantation to that permitted by L1-L6, iliac wing anatomy significantly increases procedural difficulty—a common challenge across canine models. However, bilateral pedicle screw fixation requires extensive dissection of the paraspinal muscles, which may lead to denervation atrophy and postoperative lumbar weakness syndrome. Additionally, compared with unilateral fixation, bilateral pedicle screw fixation carries a higher risk of nerve root injury [29]. However, the results of this study showed that larger angles were associated with lower strain and stress, which might be attributed to the fact that partial pedicle screws enter the cortical bone, leading to harder contact. Therefore, this could increase the risk of Grade 1 or 2 canal breach in clinical practice. Consequently, clinics should incorporate anatomical considerations to identify optimal screw trajectories while minimizing neural interference. Proper screw placement results in acceptable postoperative micromotion at fixation termini, thereby reducing displacement-related failure risks [23]. FEA can simulate pedicle screw placement on the side of nerve root decompression, theoretically reducing nerve injury by 50% after screw insertion [30].

Frankly, our study has several limitations. For example, static loading conditions may not fully reflect the complex mechanical environment of the canine lumbar spine during daily activities. Our model applied a constant 10 N vertical load to simulate standing or slow walking, but dogs experience dynamic loads that fluctuate with movement—for example, peak vertical forces can reach two to three times the body weight during running or jumping [31]. Additionally, breed-specific anatomical variability remains a critical consideration. We selected beagles as a representative medium-sized breed to minimize size-related bias; their L7 pedicles (>3 mm width) provide adequate implantation space, favoring transpedicular fixation, while lumbar spine morphology varies substantially across canine breeds. For example, large breeds (e.g., German Shepherds) exhibit longer vertebral bodies and thicker pedicles (4.5–5.0 mm in diameter) compared to small breeds (e.g., Yorkshire Terriers, pedicle diameter: 2.0–2.5 mm). These differences could affect optimal screw trajectory, as a 58° angle—identified as biomechanically favorable in beagles—might lead to pedicle breach in smaller breeds or inadequate purchase in larger breeds. Breeds with narrower L7 pedicles (e.g., Bulldogs) may also require alternative angles or screw sizes. More importantly, the model did not account for patient-specific factors such as bone quality (e.g., osteoporosis, which is common in elderly dogs), which can drastically reduce screw pullout strength and alter stress distribution [32].

## 5. Conclusions

In conclusion, the results of this study show that the peak stress fluctuates significantly between 45° and 55°, with the highest stress occurring at 50°. The stress is more stable between 56° and 60°, with the lowest stress at 58°, indicating the best biomechanical stability. Finite element fracture models developed using spinal loading scoring systems demonstrate superior clinical relevance, with simulation results showing strong concordance with biomechanical characteristics from actual cases [33]. This approach enables the quantitative assessment of vertebral injury severity, fracture fragment distribution, displacement magnitude, and kyphotic correction, all of which can be effectively simulated through finite element methods [34]. However, finite element modeling software presents considerable operational challenges, demanding substantial computational expertise. Integration of FEA with laser and/or robot-assisted guidance, or 3D printing technology, which is emerging in the veterinary field, e.g., 3D-printed personalized guidance devices capable of converting medical imaging data into anatomically accurate models, may enhance preoperative planning and surgical success rates in lumbar procedures. Future studies should systematically evaluate breed-specific implantation parameters.

## Figures and Tables

**Table 1:** Material parameters of model components.

Material	Elastic Modulus (MPa)	Poisson’s Ratio
Cortical Bone	12,000	0.30
Cancellous Bone	132	0.20
Nucleus Pulposus	1	0.49
Annulus Fibrosus	4.2	0.45
Articular Cartilage	11	0.40
Endplate	23.8	0.40
Ti-6Al-4V (Pedicle Screw)	110,000	0.30
Ti-6Al-4V (Fixation Rod)	110,000	0.30

**Table 2:** The number of division nodes and elements in the pedicle screw implantation model under different angles.

Angles	Nodes	Elements	Angles	Nodes	Elements
45°	166,209	87,002	56°	168,027	87,710
47°	165,931	86,450	57°	165,850	86,478
50°	164,932	86,269	58°	164,115	85,784
51°	164,923	86,269	59°	165,859	86,478
52°	164,923	86,269	60°	166,389	86,852
53°	166,622	86,463	63°	161,757	83,027
54°	161,725	83,010	65°	166,622	86,436
55°	168,027	87,710			

**Table 3:** Maximum total deformation values of pedicle screw implants at different implantation angles.

Angles	Maximum Total Deformation (mm)	Angles	Maximum Total Deformation (mm)
45°	0.0066	56°	0.0043
47°	0.0055	57°	0.0035
50°	0.0046	58°	0.0051
51°	0.0034	59°	0.0041
52°	0.0041	60°	0.0049
53°	0.0045	63°	0.0035
54°	0.0033	65°	0.0038
55°	0.0064		

## Data Availability

The original contributions presented in the study are included in the article material, and further inquiries can be directed to the corresponding authors.
